# Single-direction thoracoscopic lobectomy for children with congenital lung malformation: initial experience

**DOI:** 10.1186/s13019-023-02192-7

**Published:** 2023-04-28

**Authors:** Jin-Xi Huang, Qiang Chen, Song-Ming Hong, Jun-Jie Hong, Hua Cao

**Affiliations:** 1 Department of Cardiothoracic Surgery, Fujian Children’s Hospital, Fuzhou, China; 2 Fujian Branch of Shanghai Children’s Medical Center, Fuzhou, China; 3 College of Clinical Medicine for Obstetrics & Gynecology and Pediatrics, Fujian Medical University, Fuzhou, China; 4 Fujian Key Laboratory of Women and Children’s Critical Diseases Research, Fujian Maternity and Child Health Hospital, 966 Hengyu Road, Fuzhou, Fujian Province China

**Keywords:** Video-assisted thoracoscopic surgery, Lobectomy, Children, Congenital lung malformation, Single-direction

## Abstract

**Background:**

Thoracoscopic lobectomy is a common treatment for congenital lung malformation. Single-direction thoracoscopic lobectomy may be an effective and safe approach without the need to flip the lung over repeatedly, thus minimizing tissue trauma, but its use has not been reported in children. The purpose of this study was to evaluate the safety and efficacy of single-direction thoracoscopic lobectomy in children.

**Methods:**

A total of 91 patients who underwent thoracoscopic lobectomy in our hospital from January 2020 to December 2020 were retrospectively analysed. According to the inclusion criteria, 21 children were identified as the single-direction group. The details of the single-direction thoracoscopic lobectomy technique are described. Another 21 patients who underwent conventional thoracoscopic lobectomy in the same period were matched using the propensity score matching and set as the control group, the clinical outcomes between the two groups were compared.

**Results:**

The median age of the patients was 4.72 months (4.72 ± 0.90) with a mean body weight of 7.43 kg (7.43 ± 1.14). There were no significant differences in intraoperative blood loss (P = 0.549), operation time (P = 0.859), length of chest tube drainage (P = 0.102) and length of hospital stay (P = 0.636) between the 2 groups. No patients experienced bronchopleural fistula and conversion to thoracotomy in either group. All patients recovered well without respiratory symptoms or other complications after follow-up of more than 1 year.

**Conclusions:**

Our preliminary experience presented a series of single-direction video-assisted thoracoscopic lobectomy for children with satisfactory perioperative results.

## Introduction

Congenital lung malformation (CLM) is a focal developmental malformation of the lung that usually forms a single, large, multilumen, cystic structure [1]. Its incidence has been estimated to be 1:2500 [2]. Lesions associated with respiratory distress require resection, but asymptomatic lesions can be observed. The removal of asymptomatic lesions can prevent infection and progression, as well as the risk of cancer [3]. Many paediatric thoracic diseases can be treated by video-assisted thoracoscopic surgery (VATS) [4, 5], and thoracoscopic surgery is widely used mainly because of its advantages of a short perioperative thoracic drainage time, short hospital stay, and few chest wall malformations [6].

Despite the relatively safe record of VATS lobectomy, catastrophic intraoperative complications are not unheard of. A variety of operative approaches have been proposed to conduct safe, curative, and minimally invasive VATS lobectomy [7]. Single-direction lobectomy, described by Liao et al. [8, 9], has been used in lung surgery and has been proven to be safe and effective without the need to flip the lung over repeatedly, thus minimizing tissue trauma and ideally ensuring efficiency [7, 10], but its use has not been reported in children. Compared that of adults, the thoracic cavity of a child is smaller, the intercostal space is narrower, and they are also limited by one-lung ventilation and minimally invasive surgical instruments. Our approach is to perform single-direction thoracoscopic lobectomy in children with CLM, summarize and report the details that require attention during the operation, and assess the safety and effectiveness of this procedure.

## Methods

This study was approved by the Ethics Committee of our institute and followed the principles of the Declaration of Helsinki. Written informed consent was obtained from the parents of the children.

## Patients

**Fig. 1:**
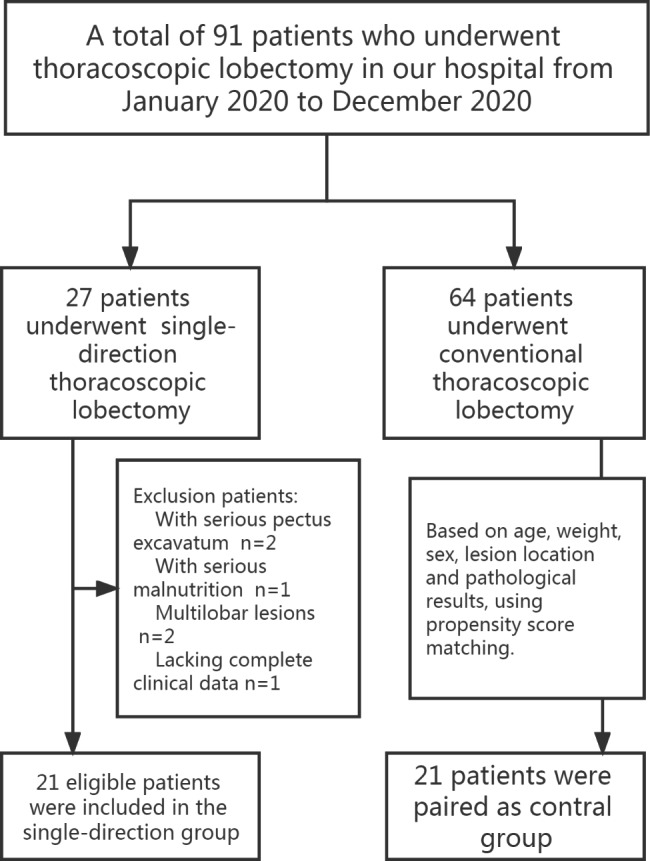
CONSORT flow diagram of participants

A total of 91 children with congenital lung malformations who underwent surgery in our centre from January 2020 to December 2020 were retrospectively studied. The inclusion criteria were as follows: 1.The operation procedure conforms to that of a single-direction thoracoscopic lobectomy. 2. Only one lobe was removed, but no surgery was performed on other lobes. The exclusion criteria were as follows: (1) Patients without complete perioperative clinical data; (2) Patients with serious complications before surgery and inability to maintain one-lung ventilation; (3) Patients with multilobar lesions. Ultimately, 21 eligible patients were included (Fig. 1). Propensity score matching was conducted for age, weight, preoperative condition, lesion location and pathological results, and additional 21 lobectomy patients underwent conventional lobectomy were ultimately matched and set as the control group. The perioperative data and postoperative complications of the two groups were analysed and compared. Baseline patient characteristics included age, sex, body weight, and lesion location. Other clinical data included operation time, blood loss, length of chest tube drainage, length of postoperative hospital stay, postoperative complications, and re-examination at 1 year after discharge. All patients were followed for at least 1 year.

## Surgical technique

The same thoracic surgical team performed consecutive surgeries on all patients. Bronchial occlusion was used for one-lung ventilation.The details of the surgical procedure of single-direction thoracoscopic lobectomy were as follows:

Left upper lobe (LUL): Retract the LUL from the ventral to the dorsal side to expose the hilum and then open the pleura with an electrocautery hook. The overlying superior pulmonary vein (PV) was carefully dissected, and the main pulmonary artery (PA) behind it was exposed to avoid subsequent injury. Since the chest cavity of children is small, endoscopic staplers are difficult to use. We ligate the superior PV with Hem-o-lock or silk thread and then transect it. Next, the main pulmonary artery is exposed. In children, the main pulmonary artery has a short trunk and branches off into the LUL artery after a short distance. We chose to isolate the branches of the LUL artery one by one and then ligate them. The LUL bronchus below can be well exposed, clipped with a Hem-o-lock and the bronchus can be transected. The oblique fissure was incised with Ligasure after the boundary of the upper and lower lungs was determined using the inflation-deflation method. The direction of lung traction is backwards throughout the excision.

Right upper lung (RUL): Similar to the LUL, the RUL is retracted from the ventral to the dorsal side to reveal the ventral hilum. First, the superior PV is dissected, and the bifurcation of the RUL vein and the middle lobe vein must be distinguished to avoid damage to the middle lobe vein. Ligate and transect the RUL vein, and then the PA is visible. While preserving the right middle pulmonary artery, the branches of the RUL artery were dissected and ligated with silk thread, and then the right upper bronchus was visible. After clipping and dissecting the RUL bronchus, the horizontal fissure was cut using Ligasure along the dilatation-collapse boundary.

Right middle lung (RML): The lung is retracted from the abdomen to the back to expose the hilum. The pleura was opened first, and then the RML vein was dissected. The RML bronchus was visible, and the PA was exposed after the bronchus was cut. RML usually has two arterial branches. Tie branches with 4 − 0 silk thread and dissect separately. The fissures are the last structures divided.

Left lower lobe (LLL) and right lower lobe (RLL):

The procedures of LLL and RLL are roughly the same; the lower lung is pulled towards the cranial and slightly ventral to expose the inferior pulmonary ligament, and the inferior pulmonary ligament is cut by an electrocautery hook and separated cephalically until the inferior PV is exposed. The inferior PV is cut off after defining its upper boundary. The LUL bronchus was dissected under the inferior PV. To avoid the principal bronchi being affected, we clamped the dorsal bronchus and the basal bronchus separately and cut them off. The dorsal artery is usually easy to identify, and the lingual PA/RML PA, superior segmental PA and basal trunk PA need to be carefully identified. After clipping and ligating the branches of the lower pulmonary artery, Ligasure was used to divide the oblique fissure along the dilatation-collapse boundary. The direction of lung traction is cephalic and slightly ventral throughout the process of resection.

After resection of the lobe, the lungs were reinflated, the remaining lungs were checked for good re-expansion and no air leakage, a closed thoracic drainage tube was placed, and the chest cavity was closed. All patients in both groups received intravenous analgesia to control postoperative pain, a standard protocol of 2.0 µg/kg of sufentanil and 100 mL of physiologic saline was used with the speed of 2.0 mL/h for the first postoperative 48 h. This protocol is equivalent to 1 µg/kg/d of sufentanil. The chest tube was removed when there was no air leakage and the daily drainage flow was less than 1 mL/kg/d. One day after the chest tube was removed, there were no signs of pneumothorax or complications in the follow-up chest radiographs, and the patient was discharged.

All statistical analyses were completed by SPSS 22.0 software (IBM Corporation, Armonk, NY, USA). Continuous data are presented as the means with SDs and medians, while categorical data are presented as patient numbers and percentages.

## Results

In this study, the median age of the patients was 4.72 months (4.72 ± 0.90) with a mean body weight of 7.43 kg (7.43 ± 1.14). The characteristics and intraoperative and postoperative variables are shown in Table 1. The mean intraoperative blood loss in the single-direction group and the control group was 10.0 ± 7.42 mL and 13.3 ± 6.40 mL (P = 0.549). The operation times of the two groups were 68.57 ± 15.42 min and 65.95 ± 15.69 min (P = 0.859). 1 patient in each group had a long period of air leakage (> 2 days), which recovered well after continuous drainage. The mean total length of hospital stay in the single-direction group and the control group was 4.23 ± 0.70 and 3.71 ± 0.90 days. There were not statistically significant differences between the two groups in terms of intraoperative blood loss, operative time, length of chest tube drainage, length of hospital stay and postoperative complications. No patients in either group had bronchopleural fistula, postoperative pneumonia, or conversion to thoracotomy, and the postoperative follow-up time was more than 1 year. No residual lesion was found in the computed tomography (CT) review that was conducted 1 year after surgery, and no recurrent pulmonary infection or other complications occurred in the patients of either group after discharge.

**Table 1:** Patients’ characteristics and outcomes

	Single-direction group	Control group	Total	P-value
Age(months)	4.75 ± 0.80	4.72 ± 0.92	4.72 ± 0.90	0.569
Weight(kg)	7.52 ± 1.07	7.43 ± 1.17	7.43 ± 1.14	0.779
Location				
Right upper lobe	2(9.5%)	2(9.5%)	4(9.5%)	
Right middle lobe	4(19.0%)	4(19.0%)	8(19.0%)	
Right lower lobe	3(14.3%)	3(14.3%)	6(14.3%)	
Left upper lobe	2(9.5%)	2(9.5%)	4(9.5%)	
Left lower lobe	10(47.6%)	10(47.6%)	20(47.6%)	
Operation time (minutes)	68.57 ± 15.42	65.95 ± 15.69	65.95 ± 15.31	0.859
Blood lost (ml)	10.0 ± 7.42	13.3 ± 6.40	13.33 ± 6.24	0.549
Length of chest tube drainage (days)	2.90 ± 0.62	2.62 ± 0.74	2.62 ± 0.72	0.102
Length of hospital stay(days)	4.23 ± 0.70	3.71 ± 0.90	3.71 ± 0.88	0.636
Postoperative complications	4(19.0%)	5(23.5%)	9(21.4%)	0.311
Subcutaneous emphysema	0	2		
Atelectasis requiring sputum aspiration	2	1		
Transient phrenic palsy	1	1		
Prolonged air leak > 2 days	1	1		

## Discussion

In order to conduct safe, curative, and minimally invasive surgery for the CLM, surgeons have developed various approaches, including the singleport method, the multi-port method, and anterior and posterior approaches. The single-direction thoracoscopic lobectomy is a simple, safe, and effective procedure for lobe resection with clear procedural steps, this method overcomes the angular limitations frequent in endoscopic procedures [8, 9]. It’s also reported that hypoplastic lung fissures were treated without difficulty and it is not necessary to dissociate pulmonary arteries within the hypoplastic lung fissure in this procedure as it is dissected as the last step in the procedure [11]. The other important reason for performing this procedure is: since each surgical method has advantages and disadvantages, it is important to master various surgical techniques, so that they can be flexibly applied according to the patient [12]. Our centre began performing single-direction thoracoscopic lobectomy in 2020. This is the first study to investigate the efficiency and safety of the single-direction approach in CLM patients. Our data showed that there were no serious postoperative complications or conversion to thoracotomy associated with this surgical method, There were no significant differences in intraoperative blood loss, operation time, length of chest tube drainage, length of hospital stay and and postoperative complications between the 2 groups, and the re-examination at one year after surgery indicated that all of the children had recovered well without residual lesions, which may indicate the safety and non-inferiority of single-direction thoracoscopic lobectomy for children with CLM.

**Fig. 2:**
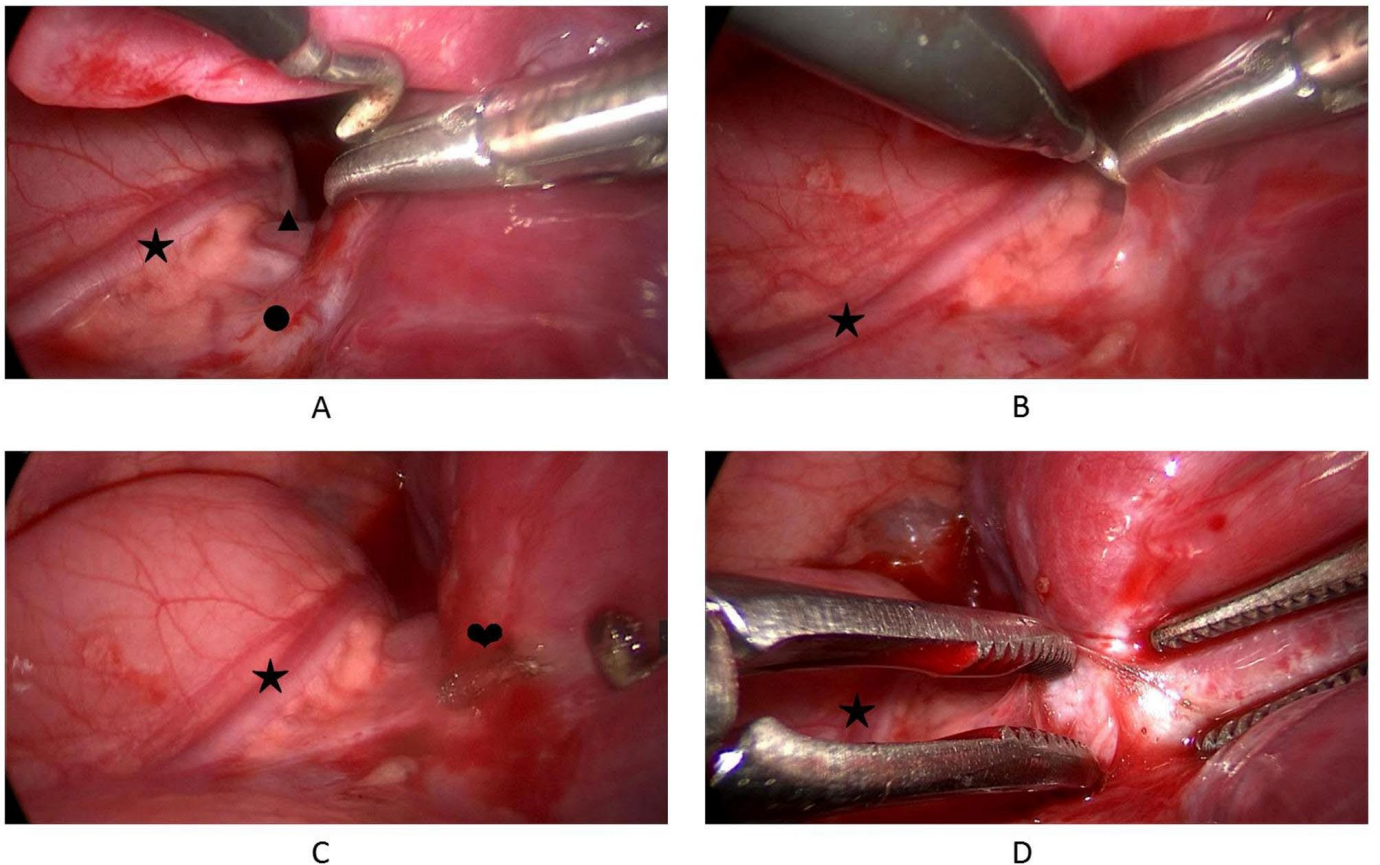
Preventing damage to the phrenic nerve during resection. A: Phrenic nerve and lung hilar. B: Lift the hilar pleura with forceps, make a small opening with electrocautery hook. C: A small opening at a safe distance from the phrenic nerve. D: Perform blunt separation to create sufficient space. (★ Phrenic nerve; ▲ Pulmonary artery; ● Superior pulmonary vein; ❤ A small opening)

Surgeons should focus on preventing damage to the phrenic nerve during resection. In addition to the direct injury of the electrocautery hook, the damage caused by heat conduction should also be considered, and a certain distance should be reserved between the phrenic nerve and the electrocautery hook. In our study, one child presented with transient phrenic nerve palsy after surgery, and postoperative chest radiography showed elevated diaphragmides on the surgical side. It decreased and returned to normal on the third day after surgery, which was considered to be related to heat conduction injury to the phrenic nerve. No other children showed symptoms related to phrenic nerve injury. Our experience has been to lift the hilar pleura with endoscopic forceps, make a small opening in the pleura away from the phrenic nerve, and perform blunt separation along this opening with the forceps to create sufficient space and avoid injury. (Fig. 2)

The vessels and bronchi in a child are treated differently than those of an adult. As mentioned above, the paediatric chest is small, and it is difficult to use an endoscopic stapler. For RUL and LUL operations, attention should be given to avoiding the use of excessive vascular clips, which may not only block the field of vision and affect subsequent operations but also compress the vessels and affect blood circulation. Ligation of two strands of silk and then transection of the vessel is the method we usually perform. However, for bronchi treatment, we recommend the use of clips, and it is worth mentioning that when clipping the bronchi, the bronchial blocker needs to be removed to avoid being clipped together.

Anatomical variations can occur in patients with CLM, in our data, one patient in control group with the dorsal bronchial absence was found by preoperative CT reconstruction, and confirmed during the operation. In another patient in single-direction group, after the right lower pulmonary artery was severed, another artery was found originated from the middle lobe artery supplying the right lower lung. Neither variations seemed to affect the course of surgery, so long as they were carefully identified.

**Fig. 3:**
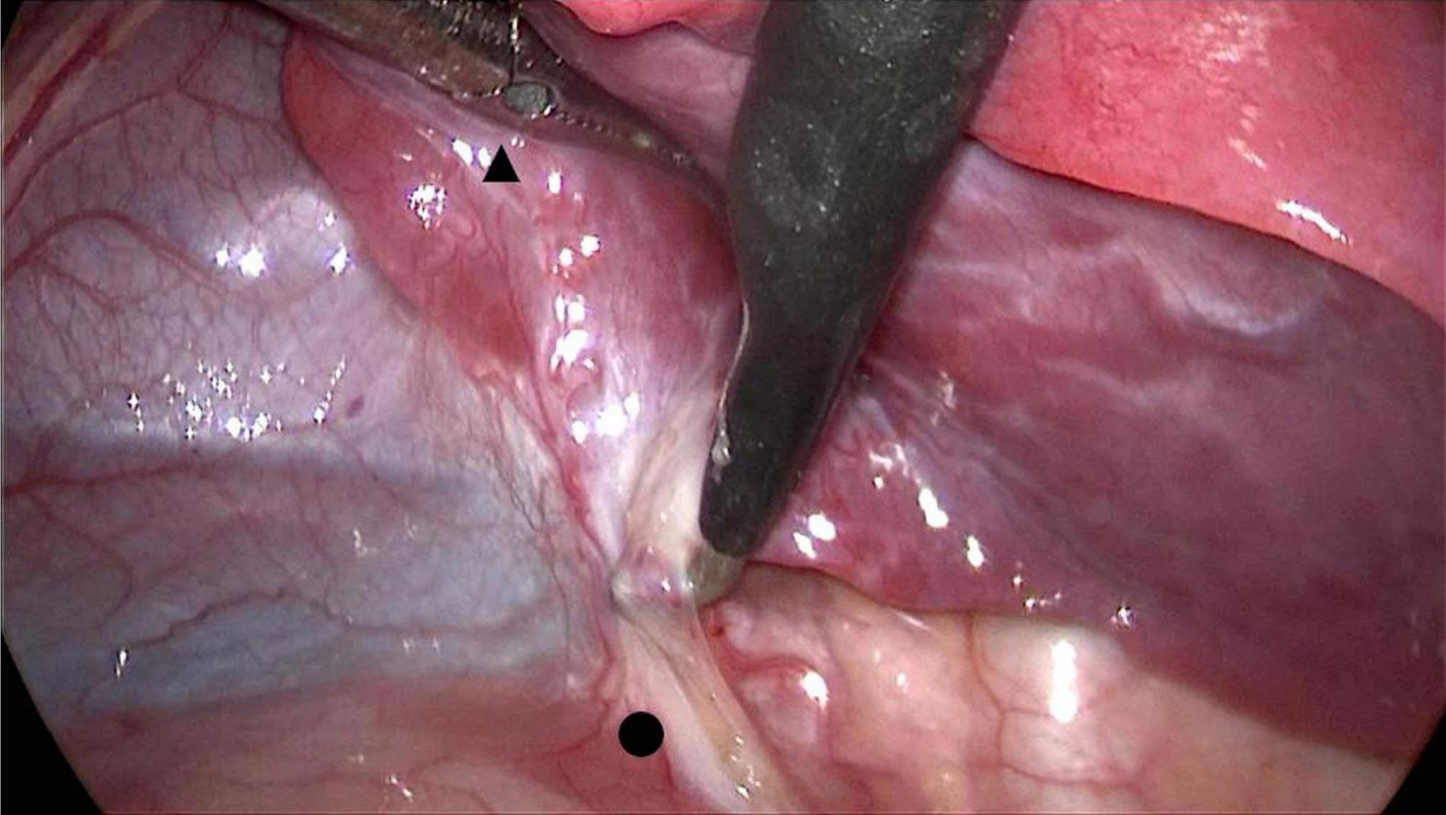
Push the lung without clamping the parenchyma. ▲ A clamped forcep; ● Inferior pulmonary ligament

The toughness of a child’s lung parenchyma is insufficient, and even if noninjury forceps or ring clamps are used to gently clamp the parenchyma, injury can easily occur. Conventional lobectomy suggests that the target artery is severed first, but single-direction lobectomy suggests that the vein is severed first. If the lung surface is injured by clamping and pulling, it is easy to conclude that when the vein is clipped, the bleeding at the injured site will gradually increase, the following procedure will be affected, and visual field exposure and tissue identification will be hindered. If the amount of blood loss increases with increasing suction, lung tissue is easily sucked into the surgical area and occludes the field of view. Our experience is as follows: When retracting the lung parenchyma, an aspirator or clamped forceps should be used to push the lung away without clamping the lung parenchyma, such as pushing the upper lung dorsally to expose the hilum or pushing the lower lung cephalically to expose the inferior ligament(Fig. 3).

There are still many limitations to our study, such as the inherent limitations of a single-centre retrospective population study. The sample was relatively small, which may limit the analytical power. Prospective studies may further examine whether there are other advantages to this surgical approach, such as advantages in the treatment of hypoplastic lung fissures. We will continue to conduct long-term follow ups of the patients and design prospective randomized trials.

## Conclusions

For children with CLM, our preliminary experience presented a series of single-direction thoracoscopic lobectomy with satisfactory perioperative results, which may indicate the safety, efficiency and non-inferiority of this approach.

## Data Availability

The data that support the findings of this study are available on request from the corresponding author. Requests to access these datasets should be directed to hjx7072@126.com.

## Abbreviations

CLM: Congenital lung malformation
VATS: Video-assisted thoracoscopic surgery
LUL: Left upper lobe
PV: Pulmonary vein
RUL: Right upper lung
RML: Right middle lung
LLL: Left lower lobe
RLL: Right lower lobe
CT: Computed tomography
